# Fipronil-induced decrease in the epididymal sperm count: oxidative effect and protection by vitamin E

**DOI:** 10.21451/1984-3143-AR2017-0040

**Published:** 2018-12-05

**Authors:** Meiriele Mazzo, Kamila Villas Boas Balieira, Paulo Francisco Veiga Bizerra, Fábio Erminio Mingatto

**Affiliations:** College of Agricultural and Technological Sciences, São Paulo State University (Unesp), Dracena, SP, Brazil.

**Keywords:** antioxidants, insecticide, oxidative stress, spermatozoids, testis

## Abstract

The toxic effects of the insecticide fipronil on the sperm production and oxidative damage in the testis were evaluated, as well as the protective action of vitamin E. Male rats received vehicle or fipronil 5 mg/kg and fipronil 5 mg/kg + vitamin E 100 mg/kg for 14 days. Thereafter, the sperm concentration in the epididymis and parameters of oxidative damage in the homogenate of testicles were assessed. Fipronil reduced epidydimal sperm count. The activity of the glutathione peroxidase enzyme increased and that of catalase was reduced in the testis. Also, a reduction in GSH and an increase in the concentration of malondialdehyde were observed in the animals treated with fipronil. The vitamin E reestablished the analysed parameters to levels similar to those of the control group. We concluded that fipronil decreased sperm production in rats because of its oxidant activity and that this effect was reversed by vitamin E.

## Introduction

Fipronil is an insecticide belonging to the family of phenylpyrazoles that acts with good selectivity in the control of insects ( Hainzl *et al*., 1998 ; Tingle *et al*., 2003 ) and is used extensively in a variety of infestations such as cockroaches, mosquitoes, termites, ants, and locusts, as well as fleas, lice, and ticks of dogs, cats, and cattle ( Chanton *et al*., 2001 ; Aajoud *et al*., 2003 ; Paim, 2010 ). Its mechanism of action is based on the non-competitive blockade of Cl^-^ channels linked to gamma-aminobutyric acid receptors (GABA), leading insects to death by neuronal hyperexcitation and paralysis ( Zhao *et al*., 2004 ).

Although it is considered safe for animals, several studies have reported that fipronil is rapidly metabolized in the liver, and its metabolites are widely distributed in tissues, especially in adipose tissue ( Tingle *et al*., 2003 ). It has been demonstrated that the formation of the sulfone metabolite, derived from an oxidation reaction, corresponds to the main route of hepatic metabolism of this compound ( Caboni *et al*., 2003 ; Tang *et al*., 2004 ). Some studies have shown that fipronil exerts a toxic effect on rat liver cells ( Tavares *et al*., 2015 ; Guelfi *et al*., 2015 ). In addition to the toxic effects on the liver, fipronil has also shown effects on reproduction, as studies have shown that the use of fipronil affects the reproductive system in females ( Tingle *et al*., 2003 ; Ohi *et al*., 2004 ; Cox, 2015), but there is a paucity of studies on fertility in males.

Infertility in males has been related to decreased viability and fertility of spermatozoa, which can be caused by excessive production of reactive oxygen species (ROS), thus exceeding the cellular antioxidant capacity and leading to oxidative stress ( Hsu *et al*., 2001 ). Oxidative stress is also considered one of the main factors associated with the loss of fertility of semen samples during their handling and storage, especially when using techniques that require the removal of seminal plasma ( Ball and Vo, 2001 ; Calamera *et al*., 2001 ; Watson, 2000 ; Aitiken and Baker, 2006 ).

Several studies have shown that vitamin E increases the concentration and quality of spermatozoa in different animal species ( Brzezinska-Slebodzinska *et al.*, 1995 ; Youself *et al*., 2003; Hong *et al.*, 2010 ; Yue *et al.*, 2010 ; Santana *et al*., 2015 ). It is believed that vitamin E is the primary component of the antioxidant system of spermatozoa ( Surai *et al.*, 1998 ), being one of the main membrane protectors against ROS and lipid peroxidation ( Akiyama, 1999 ). Its action is based on increasing the resistance of cells to hydrogen peroxide and its ability to destroy free-formed peroxides ( Droge, 2002 ).

The present study aimed to analyze the interference of fipronil in the fertility of male rats, since the existing reports of the action of fipronil on reproduction focus mainly on studies in females. The oxidative damage caused in the homogenate of the testes was considered and the protective effect of vitamin E against the toxic action of fipronil was also investigated.

## Material and Methods

### Chemicals

Fipronil [(±)-5-amino-1-(2,6-dichloro-α,α,α-trifluoro- *p*-tolyl)-4-trifluoromethyl sulfinyl pyrazole-3-carbonitrile], 96.6% purity, was kindly supplied by the company Ourofino Agribusiness (Cravinhos, São Paulo, Brazil). All the other reagents were of the highest commercially available grade. All the stock solutions were prepared using glass-distilled deionized water.

### Animals

The experimental protocols were approved by the Ethics Committee for the Use of Laboratory Animals of the São Paulo State University (Unesp), College of Agricultural and Technological Sciences, Dracena, SP, Brazil (Protocol number 23/2015). Male Wistar rats weighing approximately 200 g were used in this study. The animals were obtained from the Central Bioterium of São Paulo State University (Unesp), Botucatu, SP, Brazil, and were maintained with a maximum of four rats per cage under standard laboratory conditions with water and food provided *ad libitum*.

### Treatment of animals

The rats were randomly divided into three groups of six animals each, according to the following treatments:

Group 1 (G1) received corn oil by gastric gavage and a mixture of dimethyl sulfoxide (DMSO) and saline (0.9% NaCl) i.p.; Group 2 (G2) received fipronil (5 mg/kg body weight [BW]) dissolved in a mixture of DMSO and 0.9% NaCl i.p. and corn oil by gastric gavage, and Group 3 (G3) received fipronil (5 mg/kg BW) dissolved in a mixture of DMSO, 0.9% NaCl i.p., and vitamin E (100 mg/kg BW) dissolved in corn oil by gastric gavage. The fipronil dose used in this study corresponded to 1/20 of rat oral LD_50_ of 100 mg/kg BW ( Pohanish, 2014 ) and the vitamin E dose selection was based on previous reports in which it has been recognized as sufficient to lowering toxicity induced by others xenobiotics ( El-Demerdash, 2004 ; Yousef *et al*., 2006 ).

The period of treatment was chosen based on the results of a pilot study using the same dose of fipronil by different periods (7, 14 and 21 days) that evaluated (1) the time required to reduce sperm production; and (2) the best time for the detection of effects related to oxidative stress.

The control group for vitamin E was not performed, as in an earlier study by Santana *et al*. (2015) , it was observed that, in the same dose used in this study, Vitamin E has no effect on the reproductive and oxidative stress parameters in the testicle.

### Sperm Counting

After 14 days of treatment, the animals were euthanized by decapitation, and the testes and epididymis were collected. The cauda of the epididymis, previously cut into small pieces with scissors, was used for semen collection and the subsequent counting of sperm ( Hollenbach, 2013 ).

For the analysis of the total number of epididymal sperm, the epididymal cauda of each animal was placed in 10 mL of normal saline (0.9% NaCl) and homogenized under cooling. One hundred microliters of the resulting mash of each epididymis was placed in an individual “Eppendorf” type tube, and 900 µL of 0.9% NaCl was added to a final volume of 1 mL. The number of sperm in this solution was counted in 128 small squares of a Neubauer chamber. Counting was performed in an optical microscope with 40X magnification. The number of spermatozoa was determined using the following formula:

S = C x CF x V where: S = Sum total per animal; C = number of counted spermatozoa; CF = chamber factor (1.25) and V = dilution (10^6^).

### Homogenate preparation

The tunica albuginea and the main vessels were removed and each testis was placed in 25 mL of medium containing 250 mM sucrose, 0.2 mM EGTA, 0.1 mM EDTA, 5 mM HEPES-KOH (pH 7.4), and 0.1% bovine serum albumin (BSA), maintained at 4°C and then sliced and homogenized with a Potter-Elvehjem homogenizer. The protein concentration of the homogenate was determined using the biuret reaction with BSA as a standard ( Cain and Skilleter, 1987 ).

### Glutathione assay

The levels of GSH were determined by a fluorometric reaction with *o*-phthalaldialdehyde (OPT) ( Hissin and Hilf, 1976 ). Testis homogenate (1 mg of protein) was added to medium (125 mM sucrose, 65 mM KCl and 10 mM HEPES-KOH, pH 7.4) to a final volume of 1 mL and treated with 0.5 mL of 13% trichloroacetic acid. The mixture was stirred and then centrifuged at 9000 × *g* for 3 min. Aliquots (100 µL) of the supernatant were mixed with 2 mL of 100 mM NaH_2_ PO_4_ buffer at pH 8.0 containing 5 mM EGTA. One hundred microliters of a OPT solution (1 mg/mL) was added, and the fluorescence was measured 15 min later in a RFPC 5301 spectrofluorometer (Shimadzu, Tokyo, Japan) using 350/420 nm as the excitation/emission wavelength pair. The data are expressed in nmol/mg protein estimated using a standard curve.

### Membrane Lipid Peroxidation (LPO) assay

The level of LPO was estimated by malondialdehyde (MDA) generation ( Buege and Aust, 1978 ). The testis homogenate (5 mg of protein) was added to a tube. Following the addition of 0.2 mL of 8.1% SDS, 1.5 mL of 20% acetic acid and 1.5 mL of 0.67% thiobarbituric acid (TBA, aqueous solution), glass-distilled deionized water was added to a final volume of 4 mL. The mixture was incubated for 60 min at 85°C. The MDA-TBA complex was extracted with 5 mL of n-butanol and the absorbance was measured at 535 nm in a NI 2000 UV spectrophotometer (NOVA Instruments, Piracicaba, SP, Brazil). The MDA concentration was calculated with ε = 1.56 × 10^5^ M^-1^ cm^-1^.

### Oxidation of protein thiol groups

The concentration of thiol (-SH) groups of proteins was determined using the Ellman reagent according to Sedlak and Lindsay (1968) with some modifications. A sample of the homogenate (5 mg protein) was treated with 1 mL of 5% trichloroacetic acid (TCA) containing 5 mM EDTA and subjected to centrifugation at 2500 *g* for 5 minutes. The protein precipitate was washed twice with the same TCA-EDTA solution. The proteins were redissolved in 3 mL of 0.1 M Tris-HCl buffer, pH 7.4, containing 5 mM EDTA and 0.5% sodium dodecyl sulfate. Aliquots of this solution were treated with 0.1 mM 5,5´-dithiobis(2-nitrobenzoic) acid (DTNB) dissolved in 2 mL of Tris-EDTA buffer, pH 8.6. The samples were incubated in the dark, and the absorbance was measured at 412 nm in a DU-800 spectrophotometer (Beckman-Coulter, Fullerton, CA, USA) and the values subtracted from a “blank” obtained by treating the samples with 5 mM N-ethylmaleimide prior to the reaction with DTNB. The concentration of thiol groups was determined using the molar extinction coefficient of 13,600 M^-1^ cm^-1^.

### Parameters of active oxidative stress in the supernatant obtained from the testicular homogenate

For the determination of the activity of the enzymes glutathione peroxidase (GPx) and catalase (CAT), 1.5 mL of the homogenate was added in a 2 mL Eppendorf tube, and this sample was centrifuged at 8500 *g* for 10 min. The supernatant protein was determined using the biuret reaction, according to Cain and Skilleter (1987) , using BSA as standard.

### Glutathione Peroxidase activity

The activity of glutathione peroxidase (GPx) was determined by an indirect method based on the oxidation of GSH to GSSG, with the consequent oxidation of NADPH catalyzed by glutathione peroxidase ( Flohé and Günzler, 1984 ). The reaction system was composed of 1.5 mL containing: 1.0 mM GSH, 0.2 mM NADPH, 0.25 mM H_ 2_O_2_, 0.5 mM EDTA, and 0.10 M sodium phosphate buffer (pH 7.6), 0.1% Triton X-100 and 50 μL of the testicular homogenate supernatant. After incubating the samples at 30°C for 5 minutes, 10 µL of 20 mM NADPH was added, and the variation in absorbance was determined at a wavelength of 340 nm in a DU-800 spectrophotometer (Beckman-Coulter, Fullerton, CA, USA). The oxidation of 1 µmol NADPH/min was used as a unit of GR. The specific activity was expressed as units per mg of protein.

### Catalase activity

The evaluation of catalase enzyme activity was performed with 50 μL of the supernatant of the testicular homogenate in 1.75 mL of potassium phosphate buffer (50 mM, pH 7). The reaction was started by adding 200 μL of 10 mM H_2_O_2_. Catalase activity was defined as the amount of enzyme required to decompose 1 nmol of H_2_O_2_ per minute at 25°C. The variation in absorbance was determined at a wavelength of 230 nm in a DU-800 spectrophotometer (Beckman-Coulter, Fullerton, CA, USA). The specific activity was expressed as units per mg of protein (Aebi and Bermeyer, 1974).

### Statistical analysis

Significant differences were calculated by one-way analysis of variance (ANOVA) followed by the Tukey test using the GraphPad Prism software, version 4.0 for Windows (GraphPad Software, San Diego, CA, USA). Values of P < 0.05 were considered significant.

## Results

### Effect of fipronil on the epidydimal sperm count

**Figure 1:**
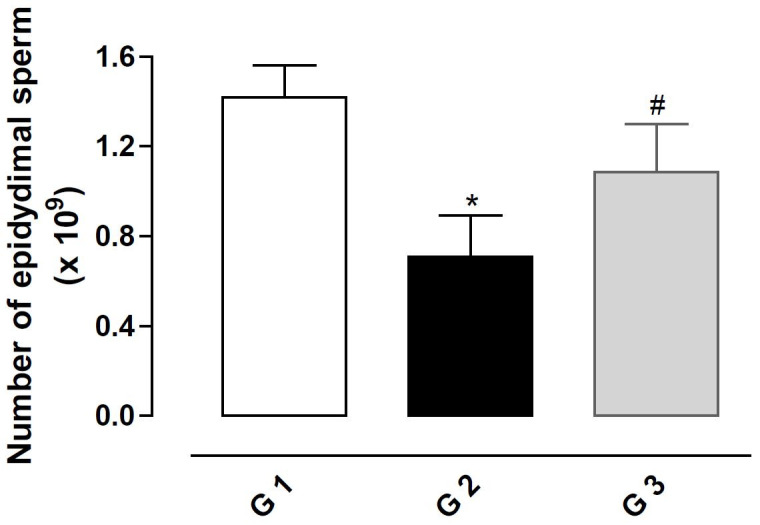
Number of sperm in the epididymis of rats exposed to fipronil and the protective action of vitamin E. The results represent the mean ± SEM of six animals per group. G1 = control; G2 = fipronil 5 mg/kg BW; G3 = fipronil 5 mg/kg BW + vitamin E 100 mg/kg BW. *Significantly different from control (G1; P < 0.05). ^#^Significantly different from the group treated with fipronil (G2; P < 0.05).

The treatment with fipronil 5 mg/kg BW (G2) decreased the sperm count significantly in the cauda epididymis in comparison with the control group (G1) (P < 0.05; Fig. 1 ). The simultaneous treatment with fipronil and vitamin E 100 mg/kg BW resulted in counts similar to control values (G3).

### Effect of fipronil on the oxidative status of glutathione and thiol groups of proteins

**Figure 2:**
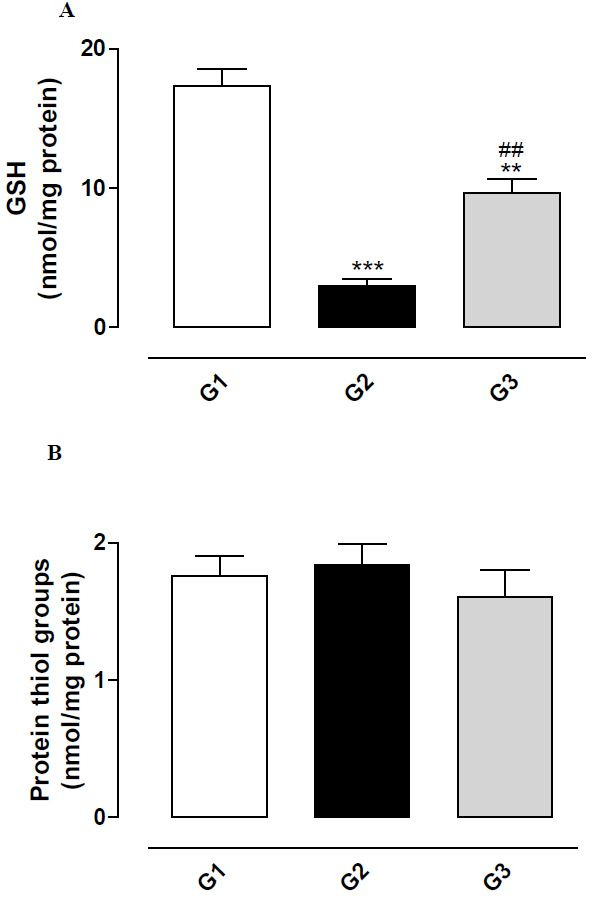
Concentration of reduced glutathione (GSH) (A) and thiol groups of proteins (B) in testis homogenate from rats exposed to fipronil and the protective action of vitamin E. The results represent the mean ± SEM of six animals per group. G1 = control; G2 = fipronil 5 mg/kg BW; G3 = fipronil 5 mg/kg BW + vitamin E 100 mg/kg BW. **^,^***Significantly different from control (G1) (P < 0.01 and P < 0.001, respectively). ^##^ Significantly different from the group treated with fipronil (G2) (P < 0.01).

Administration of fipronil (G2) induced a significant reduction in GSH concentration in the testis homogenate (P < 0.001; Fig. 2A ). The simultaneous treatment with fipronil and vitamin E (G3) had a protective effect on this oxidation.

No significant reduction was observed in the concentration of thiol groups of proteins in the testis homogenate of the fipronil-treated group (G2; Fig. 2B ), showing that fipronil did not promote the oxidation of these groups.

### Effect of fipronil on membrane lipid peroxidation

**Figure 3:**
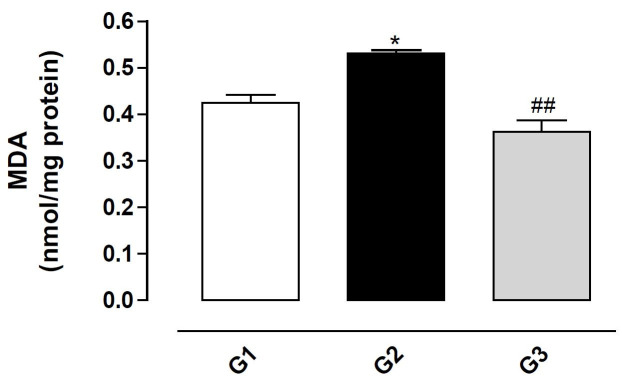
Concentration of malondialdehyde (MDA) in the testis homogenate from rats exposed to fipronil and the protective action of vitamin E. The results represent the mean ± SEM of six animals per group. G1 = control; G2 = fipronil 5 mg/kg BW; G3 = fipronil 5 mg/kg BW + vitamin E 100 mg/kg BW. *Significantly different from control (G1; P < 0.05). ^##^Significantly different from the group treated with fipronil (G2; P < 0.01).

The peroxidation of membrane lipids was assessed by the measurement of malondialdehyde (MDA). The results demonstrated that treatment with fipronil (G2) significantly increased the concentration of MDA (P < 0.05; Fig. 3 ), and that the use of vitamin E (G3) significantly reduced the effect of fipronil (P < 0.01), indicating protection against the harmful effects of fipronil on the lipids.

### Effect of fipronil on glutathione peroxidase and catalase activity

**Figure 4:**
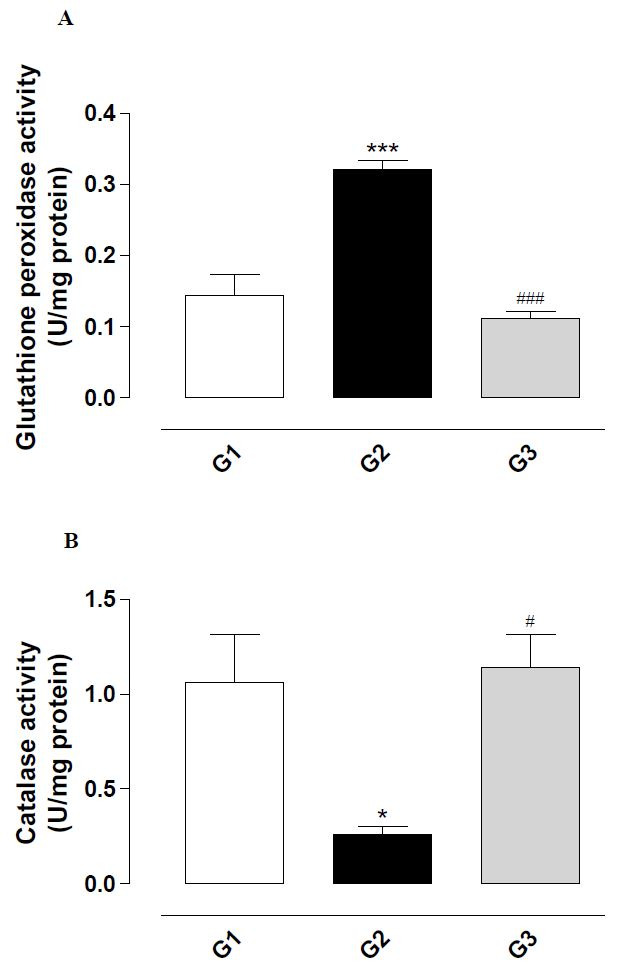
Activity of the enzymes glutathione peroxidase (A) and catalase (B) in the supernatant of testis homogenate from rats exposed to fipronil and the protective action of vitamin E. The results represent the mean ± SEM of six animals per group. G1 = control; G2 = fipronil 5 mg/kg BW; G3 = fipronil 5 mg/kg BW + vitamin E 100 mg/kg BW. *^,^***Significantly different from control (G1; P < 0.05 and P < 0.001, respectively). ^#,###^ Significantly different from the group treated with fipronil (G2; P < 0.05 and P < 0.001, respectively).

The glutathione peroxidase activity was significantly increased in the group treated with fipronil (G2) compared with the control (G1; P < 0.001; Fig. 4A ), and the simultaneous treatment of the animals with fipronil and vitamin E (G3) prevented the increase in glutathione peroxidase activity (P < 0.001).

Fipronil reduced significantly the catalase activity (G2) when compared with the control (G1; P < 0.05; Fig. 4B ), and the simultaneous treatment of the animals with fipronil and vitamin E (G3) prevented the reduction in the enzyme activity (P < 0.05).

## Discussion

The use of chemical pesticides is widespread throughout the world. Animals and humans are exposed to pesticides through a number of different sources: residue on food, contaminated tap water, occupational exposure, repellents, household use and application against fleas and ticks ( Epa, 2017 ).

Fipronil is a pesticide that belongs to the phenylpyrazole chemical group. It is an insecticide with widespread use in the control of many agricultural and domestic pests ( Tingle, *et* al., 2003 ). There are several cases in the literature of animal and human poisoning due to its intentional ingestion, accidental exposure, or incorrect use. The effects of fipronil on fertility in females also have been reported in the literature ( Tingle *et al*., 2003 ; Ohi *et al*., 2004 ; Cox, 2015). However, there are few studies evaluating its effect on fertility in males.

Barros *et al*. (2016) evaluated the effects resulting from perinatal exposure to fipronil at concentrations of 0.03, 0.3, or 3 mg/kg BW and their possible late repercussions on reproductive parameters in male rats. The results demonstrated that perinatal exposure to fipronil has long-term effects on sperm motility and that the epididymis may be a target organ. The aim of this study was to investigate the effects induced by orally (gavage) administered fipronil on fertility in male rats, the involvement of oxidative stress, and the protective effect of vitamin E.

The epididymis has an important role in sperm maturation, protection, transport, concentration and storage and has been considered to be highly susceptible to the damage induced by ROS because of their high content of polyunsaturated fatty acids ( Noblanc *et al*. 2011 ). To counteract the effects of ROS, epididymis is equipped with antioxidant defense systems, which prevent damage to spermatozoa ( Park *et al*. 2012 ). Treatment with fipronil 5 mg/kg BW led to a decrease in the total number of spermatozoa in the cauda of the epididymis of rats, an effect reversed by treatment of animals with vitamin E, indicating that an oxidizing activity of fipronil may be involved.

According to Halliwell and Gutteridge (2007) , physiologically, the organism can defend itself against reactive oxygen species (ROS) using its reserves of antioxidant enzymes, among them glutathione peroxidase (GPx), glutathione reductase (GR), and catalase (CAT). Oxidative stress is the imbalance between ROS and antioxidant defense, including decreased GSH levels, a reduction in the activity of antioxidant enzymes and the consequent lipoperoxidation ( Sies, 1986 ).

In the present study, treatment with fipronil 5 mg/kg BW resulted in a decrease in GSH concentration in the testis, and treatment with vitamin E increased the concentration of this tripeptide, showing protective effect. These results are in accordance with the work of Mossa *et al*. (2015) that demonstrated a reduction in GSH concentration in the liver and kidney of rats treated with fipronil at a dose of 10 mg/kg BW. Badgujar *et al*. 2015a observed a decrease in GSH concentration in the kidney and brain of mice treated with fipronil (5 and 10 mg/kg BW), and vitamin E could protect against the oxidizing effect of fipronil. Another study conducted by the same group of researchers with fipronil at doses of 5 and 10 mg/kg showed a decrease in the concentration of GSH in the liver of mice and also a protective effect of vitamin E ( Badgujar, *et al*., 2015b ).

The oxidation of proteins mainly results from the action of free radicals on the thiol group (-SH) of the cysteine, also causing aggregation and fragmentation of amino acids, which leads to protein denaturation ( Arkezenov and Markesbery, 2001 ; Leichert and Jakob, 2004 ). Some of the consequences of protein oxidation are the reduction or inactivation of enzymatic activity and damage to receptors, signal transduction pathways, and transport ( Halliwell and Gutteridge, 2007 ). However, in this study, the treatment of the animals with fipronil did not alter the concentration of protein thiol groups, indicating that its effect on proteins is not related to the oxidizing action on this group.

There was an increase in the formation of MDA, an indicator of lipoperoxidation, in the testicular homogenate relative to the control group; however, concomitant treatment with vitamin E protected lipids from oxidation caused by fipronil. These results are consistent with the studies of Badgujar *et al*. (2015a) , in which fipronil caused lipoperoxidation in the kidney and brain of mice, and of Badgujar *et al*., 2015b who observed increased lipoperoxidation in the liver of mice. Both studies also demonstrated protection by vitamin E, in agreement with our results.

Fipronil promoted an increase in the activity of the GPx in the testis homogenate in comparison with the control group, whereas vitamin E stabilized the enzyme activity at normal levels. The biological function of GPx is to reduce hydrogen peroxide to form water ( Ribeiro *et al*., 2015 ). The increase in GPx activity found in this study disagrees with that described by Mossa *et al*., (2015) . In analyses of liver and kidney of rats treated with fipronil at doses of 0.1, 1.0, and 10 mg/kg BW, they demonstrated a decrease in the activity of GPx caused by the higher concentration of the insecticide. Such an increase in the activity of antioxidant enzymes has been attributed to the defense mechanism against oxidative stress in an attempt to repair cell damage ( El-Gendy *et al*., 2010 ).

Catalase is a major primary antioxidant defense component and, together with the GPx, is involved in the catalysis of hydrogen peroxide decomposition into water. Decreased catalase activity implies Haber Weiss or Fenton reaction-mediated conversion of hydrogen peroxide to the hydroxyl radical, which is one of the most reactive and short-lived biological radicals ( Reed, 2001 ). In the present study, there was a decrease in the activity of the CAT in the testis of rats treated with fipronil relative to the control, and vitamin E had a protective effect. This result agrees with the study of Badgujar *et al*., (2015a) in which a reduction in CAT activity was observed in the liver of mice at a dose of 5 mg/kg BW by oral gavage. The authors attributed the decrease of the CAT activity to the down-regulation effect of fipronil in the mRNA expression of the enzyme. In this same study, prior administration of vitamin E reversed the effect of fipronil.

Therefore, the results obtained in this study demonstrate that fipronil decreases the production of spermatozoa, which could lead to infertility in male rats and the mechanism of toxicity of the insecticide on the testicles may be related to the induction of oxidative stress. They also suggest that vitamin E may be an efficient alternative in preventing damage caused by fipronil. Although in the present study the effect of fipronil in the oxidative stress has been evaluated only on the testicle homogenate, such effects can also be expected for the epididymis, since it is also susceptible to the effect of ROS.
